# “It is the medicines that keep us alive”: lived experiences of diabetes medication use and continuity among adults in Southeastern Tanzania

**DOI:** 10.1186/s12913-015-0768-5

**Published:** 2015-03-18

**Authors:** Emmy Metta, Hinke Haisma, Flora Kessy, Eveline Geubbels, Inge Hutter, Ajay Bailey

**Affiliations:** Ifakara Health Institute, P.O. Box 78373, Dar es Salaam, Tanzania; Population Research Center, Faculty of Spatial Sciences, University of Groningen, Landleven 1, 9742 AK Groningen, The Netherlands; Mzumbe University, P.O. Box 20226, Dar es Salaam, Tanzania

**Keywords:** Diabetes, Medication use continuity, Access, Rural areas and Tanzania

## Abstract

**Background:**

Diabetes is a chronic condition which requires many patients to use medications for the remainder of their lives. While this regimen is demanding, little research has been done on the experiences individuals have with diabetes medication use and the continuity of use, especially patients from rural areas of Tanzania. This study explores the lived experiences of diabetes medication use and the continuity of use among adult diabetes patients from rural communities with limited access to diabetes medicines.

**Methods:**

We conducted 19 in-depth interviews to explore patients’ experiences with diabetes medication use and the continuity of use. We employed the 5As of access to care to situate the behavioral practices surrounding diabetes medication use in the study settings. The data analysis followed grounded theory principles, and was conducted with the help of NVivo 9.

**Results:**

Study participants expressed positive attitudes toward the use of diabetes medicines, but also concerns about affordability. The patients employed two main strategies for dealing with the cost. The first was to increase their available funds by spending less money on family needs, selling household property, asking family and friends for money, or borrowing cash. They also reported sourcing medicines from pharmacies to save on consultation and laboratory costs. Second, participants reported using less than the recommended dosage or skipping doses, and sharing medicines. The geographic accessibility of diabetes service providers, the availability of medication, and the organization of the diabetes services were also cited as barriers to taking medications and to using them continuously.

**Conclusions:**

The strategies employed by the people in this study illustrate their resilience in the face of poverty and failing health care systems. More comprehensive strategies are therefore needed to encourage consistent medication use among people with chronic conditions. These strategies could include the reduction of prices by pharmaceuticals, the strengthening of community risk-pooling mechanisms and sustained health campaigns aimed at patients and the community.

## Background

Diabetes is a global pandemic, and increasingly affects the people of sub-Saharan Africa (SSA) [1,2]. The increasing prevalence of diabetes among other non-communicable diseases (NCDs) is shaped by the demographic and the epidemiological transition, which is characterized by rapid urbanization [2], the aging of the population, and changes in behavioral and dietary practices associated with economic development [1,3,4]. Type 2 is the most common type of diabetes. It usually occurs in adults, but is increasingly observed in children and adolescents as well [1]. More than half of all diabetes cases in Africa are in Tanzania, South Africa, Nigeria, and Ethiopia [1]. Tanzania alone had more than 1.7 million people with diabetes in 2013. Type 2 diabetes is associated with overweight, and weight reduction through diet or exercise or in combination with medication can help to control the condition [5]. Many diabetes patients have to take medication for the rest of their lives to improve their insulin uptake and to prevent or delay the development of complications and premature death [1]. For rural areas of SSA, especially of Tanzania, information about the behavioral experiences related to long-term medication use among NCD patients has so far been limited.

The main challenge in managing diabetes in SSA is providing accessible care and appropriate medications to patients who need them [6]. The delivery of diabetes services and the resources for managing the condition compete with the need to combat infectious conditions such as HIV/AIDS, tuberculosis, and malaria [2,6-8]. In response to the scarcity of resources and the disease patterns, SSA health systems have focused on providing episodic care for acute symptomatic conditions [9,10] and services for maternal, infant, and child health [7]. The episodic model of care is not suited to managing NCDs, which involves continuing care [11,12]. Many people with NCDs, including diabetes, are unable to obtain continuous appropriate care [6,8,13] due to problems related to quality and access [3,6,8,14]. Lack of access to continuing diabetes care is associated with poor health outcomes and/or premature death [15]. However, there is a lack of in-depth knowledge about how people with diabetes in SSA manage their prescribed medicines. This information can be used to better plan and tailor interventions for facilitating continuity of care for patients with chronic conditions.

**Figure 1:**
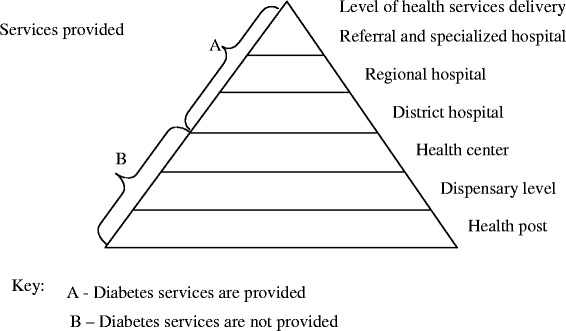
**Structure of the Public health service delivery in Tanzania.**

In Tanzania, the establishment in 2003 of a network of diabetes clinics markedly improved the access to and provision of diabetes treatments and services. Before the network was created, diabetes services were offered at regional hospitals through outpatient care by general practitioners, and at specialized referral hospitals [16]. Although access to diabetes services has improved, these services are mainly provided at the district, regional, and referral hospitals levels (see Figure 1), and are thus primarily available in urban areas.

According to a recent national survey, diabetes services were available in 20% of urban and 9% of rural health facilities, but only half of these facilities had the diagnostic capabilities, the drugs, the medical personnel, and the guidelines needed for diagnosis and patient management on the day of the survey [17]. Systematic reviews of diabetes medication use have reported that non-adherence [18-20] is common. This paper explores how access to diabetes services in rural areas shapes individuals’ behavior and their experiences regarding medication use and continuity.

### Access as a concept

The concept of access has been defined in different ways [21-23]. This study will use the definition by Peters et al. [23] “the timely use of service according to the need” [23]. Access to care becomes one of the major concerns when time is an important factor in the utilization of health services [24]. Penchnasky and Thomas 1981 defined five specific areas, referred to as the 5As of access, to indicate the *“degree of fit between the patient and the health care system”* [22]. The 5As of access to care (see Table 1) are among the dimensions which influence the initiation and utilization of health services when and where the services are needed [23,24].

**Table 1:** **The 5As access to care dimensions**

**Dimensions of access**	**Description**
Acceptability	The relationship between clients’ attitudes about the service characteristics
Affordability	The relationship between the price of the services and the clients’ ability to pay
Accessibility	The relationship between the location of the service and the location of the clients taking into account client transportation resources, travel time, distance, and cost
Availability	The relationship between the amount and the type of the existing health services and the clients’ needs
Accommodation	The relationship between the manner in which the services are organized and clients’ expectations

Source: Penchnasky and Thomas 1981.

The literature has shown that the majority of diabetes patients in most resource-poor countries are dying because they cannot access insulin [13,25,26]. The availability of diabetes medications [13,14,27] and their affordability [2,25,28] are the main barriers to medication use and continuity among diabetes patients. Lack of access to these treatments, service disparities between urban and rural areas, high costs, perceptions of symptoms, and satisfaction with the quality of services provided are among the obstacles to diabetes self-care, including to continuity of medication use [15,29].

Although the access to care dimensions are well-suited to and have been widely used in assessing barriers to health care service utilization in low-income countries [23,24,30] and elsewhere [21,31], in this article we use the dimensions to situate the behavioral practices surrounding diabetes medication use and continuity in a rural setting. Only a few existing studies have looked at the medication use experiences of patients, especially of those with non-communicable diseases such as diabetes. The current study, which aims to fill this gap, is timely because SSA countries, including Tanzania, are experiencing rising burdens of non-communicable conditions. Thus, there is a need to examine continuity in medication use.

## Methods

### Study setting

The study was conducted in the village of Viwanjasitini and at the diabetes clinic in the Kilombero district in the Morogoro region of Tanzania. Viwanjasitini, a semi urban village, is one of 102 villages in the Kilombero district. The village had a total of 12,823 inhabitants (6,359 men and 6,464 women). The village was included in the study in order to gain access to people living in the community who were diagnosed with diabetes.

The only diabetes clinic in the district, situated on the premises of the designated district referral hospital, is less than five kilometers away from the village. The clinic provides diabetes services to residents of the district and of the neighboring areas. The clinic does not provide medications, but instructs patients to buy them from the hospital pharmacy. The costs of the medications are covered by the patients themselves or by health insurance, which is generally available to government employees only. The diabetes clinic is staffed by three clinicians (a consultant physician, and two assistant medical officers), a nurse assistant, a nursing officer, and two medical attendants. At the time of the study, more than 497 diabetes patients were registered at the clinic and were scheduled for routine monthly visits for the monitoring of their condition and the refilling of their diabetes medications. The clinic was included in the study in order to gain access to people who were living in remote villages. This study was part of a bigger qualitative study on health care-seeking behavior among patients with diabetes or malaria. In this paper, we present results from the interviews on diabetes, as we are interested in exploring the lived experiences of individuals.

### Study design and recruitment of participants

A qualitative research design employing in-depth interviews was chosen in order to collect information on personal experiences related to the continuity of diabetes medication use. The interviews were conducted by a team of three social scientists, including the first author and two trained research assistants. The study participants were adults who had been diagnosed with diabetes more than six months prior to the time of the study. The participants from the semi-urban village were purposively selected with the help of the village leaders. The researchers visited household in the village, asking whether anyone in the household had diabetes. To avoid recruiting people of the same social network, different entry points were used. If an individual reported that he or she had a diabetes diagnosis, his or her clinic or medical card was reviewed for confirmation. Participants from the remote villages were selected from among the clinic attendees with the help of a clinic nurse. During a visit to the clinic, the researcher—in consultation with the clinic nurse—purposively identified individuals who came from rural areas, after the patients were registered and their blood pressure and weight were measured. The participants recruited through the clinic were paid a small amount to reimburse them for their transportation costs. The recruitment of participants at both sites ended after data saturation was reached.

### Data collection and analysis

Data collection was conducted between February and March 2013. In-depth interviews were conducted in Swahili with 19 adult diabetes patients. Of these patients, nine were male and 10 were female. The in-depth interview guides, which were refined following the analysis of the pilot study, were applied during the interviews. The interviews were 45–60 minutes long. The transcripts were anonymized and crosschecked for quality by the first author before they were imported to NVivo 9 (QSR International Pty Ltd, Australia). All of the transcripts were analyzed in their original language. The analysis process had two levels. The first level involved developing inductive and deductive codes. The second level involved categorizing the codes into themes and family codes following principles of grounded theory. After linking the emerging concepts and the 5As access to care dimensions, the following themes emerged: (i) perceptions of the chronicity of diabetes and of the acceptability of medications, (ii) affordability, (iii) accessibility, and (iv) availability and accommodation.

### Ethical approval

The study was approved by institutional review boards of the Faculty of Spatial Sciences, University of Groningen in the Netherlands; the Ifakara Health Institute (IHI) in Tanzania; and the National Tanzanian Medical Research Co-coordinating Committee of the National Institute for Medical Research (NIMR). Each participating individual provided a verbal consent to participate.

## Results

### Perceptions of the chronicity of diabetes and of the acceptability of medications

The participants demonstrated that they understand that diabetes is a chronic disease, with several stating that diabetes is not a curable disease, but a lifelong condition:

*“…Diabetes is not a curable disease. Rather, the medicines we are using are just for relief; that is what we were told at the clinic one day.”* Female 03, 58 years^a^

As they were aware of the chronicity of diabetes, participants expressed despair and hopelessness about regaining their level of health prior to the onset of diabetes:

*“….These medications are not a cure… they only reduce the severity of the disease so that one can continue living… being cured from this disease is a dream that will never be realized.”* Female 13, 40 years

Most participants nonetheless had positive views of diabetes medications, citing their benefits:

*“…I mean when you are taking the medications you don’t feel a difference…. you feel normal…. eeh I mean for those of us with diabetes it is the medicine that keeps us alive …if you use your medicine as instructed.... you will have energy and may perform your activities as usual.. no one will know what you are suffering from…”* Male 04, 52 years

In addition to having a positive opinion of diabetes medicines, some participants reported using local herbs, such as Moringa oliferus, aloe vera, lime leaves, and herbal mixtures. Information from relatives/colleagues and/or advertisements from local herbalists on the effectiveness of herbal treatments in managing the condition were cited as motivations for using them. Other participants expressed doubts about the long-term use of the diabetes medications, and some noted that taking the medicines while drunk and/or on an empty stomach can have negative effects. Forgetting to take medicines when the condition is less severe has been shown to contribute to interrupted use. Overall, the study participants acknowledged that taking their diabetes medications continuously is the best way to manage their condition. Among the main reasons participants gave for taking their medications as prescribed was the reduced severity of their symptoms, such as frequent urination, fever, and fatigue. While the participants accepted the medicines and were willing to use them, lack of access remained a barrier to continuous use.

### Affordability

Several participants said they were unable to afford their consultation, laboratory, and medication costs. On average, diabetes patients reported monthly costs of Tshs 5,000 (USD 3.2) for consultation and laboratory tests, and of Tshs 4,600 – Tsh 52,000 (USD 2.9 – 33.7) for medicines, depending on whether they were using oral hypoglycemic medications or insulin. The minimum monthly wage of a public employee in Tanzania is Tshs 265,000 (USD 171.521). However, based on estimates of monthly expenditures made by researchers working in the field, the average monthly income per person in the district is Tshs 14,946 (UDS 9.6). Having to rely on erratic sources of income, such as income from subsistence farming, made paying for the medications especially difficult:

*“….Most of us are jobless; we depend on farming to earn a living. We use the hand hoe and depend on whether it rains or not. If it rains you are thankful because you can harvest something to sell and get enough money for even a half dose. If it does not rain you get nothing…. and there is nothing you can do.”* Female 15, 36 years

Under the cost-sharing policy in Tanzania, individuals with chronic diseases like diabetes are exempted from paying for medical treatments at public health facilities. However, because of the poor quality of public services—and because public services are unavailable in most areas of the country—these individuals had to use private health services, and thus had to cover the costs themselves or to go without medications.

People who lived in remote villages reported finding the high cost of medications and related services especially challenging. When they needed to refill their prescription, they had to cover not only the consultation and laboratory costs, but also the costs of transport, lodging, and food:

*“I need to have enough money for a return transport, accommodation, and food before I can think about going to the clinic—and that doesn’t include the money for laboratory tests and medicines… my financial resources are low…. I just leave it to God—if I have money I go to get the medicines, and if I don’t, I stay home and go without….”* Female 06, 41 years

The participants reported using several strategies for getting the money they needed to adhere to their medication regimen. To pay for diabetes services, some participants said they spent less money on family needs, such as sending children to school. In some cases, people had to sell assets, which then pushed them and their families further into poverty:

*“….Because …you need medicines…. you have to sell whatever you have in the household to meet medical-related costs ….and in most cases the money you get… buys some medicine ….but only enough for a certain amount of time. Then do you think it will be easy to replace what you sold? …What will you do after you sell everything? …With this disease you need enough money to pay for medicine until the day you go to the grave.”* Male 08, 57 years

To avoid the costs associated with clinical consultation and laboratory tests, some participants reported buying medications directly from pharmacies. To obtain medications from a pharmacy, patients presented their prescription book, which contains the relevant medical and drug histories.

A number of participants also reported asking family members for financial support, including husbands, wives, and children; while some reported borrowing cash from friends. In addition, some participants said they decide whether they need to take their medication, or whether they can skip it, based on a self-evaluation of their condition:

*“…We are supposed to use these medicines every day, but when I look at the real situation…. I wonder where the money for buying enough medicine to use every day would come from. If I feel a little better on a certain day I keep the medicine so that I can use it on another day.”* Male 11, 73 years

Participants reported using less than the recommended dosage to delay having to refill a prescription:

*“…Medicines are very important for treating this disease…That is why you find people using a half of the dose and saving the rest for the days ahead, because you do not know how it is going to be tomorrow*… [Have you experienced that situation?] *Yes…sometimes instead of injecting myself twice in a day I inject myself only once… At least when I do this the medicine for a month can last me two months.”* Male10, 75 years

Some participants said they went without medications when they could not afford them. Others reported sharing their diabetic medicines with other diabetes patients in the neighborhood when one of them ran out. This type of sharing appears to reduce the risk that an individual will miss a dose.

### Accessibility

Inadequate access to diabetes medications and services appears to have negatively affected medication use and the continuity of use among the participants, especially among those living in remote villages. Residents of the district have access to diabetes services at the district diabetes clinic of the district referral hospital (see Figure 1), located in Ifakara town. Thus, the participants who were living in remote villages reported having to travel to town to access the services. Lack of a reliable means of transport, long waiting and journey times, and high transport costs affected access to care. To save on transportation and related costs, some participants admitted giving their diabetes book and money to buy medications to people who were going to town. Travel time and costs were reported to double during the rainy season, a period when some people were unable to access care of any kind:

*“….During the rains it is very difficult to come to the clinic because our roads are impassable, and cars are unable to reach the village….. This is why even I could not come to the clinic last month…”* Male 09, 58 years

### Availability and accommodation

The availability of diabetes services, including medicines, was cited as another barrier to continuous use. Although lack of money appeared to be the main barrier to accessing these medicines, the participants also mentioned that the medicines were not always available at lower-level health facilities (see Figure 1), and that the supply of medications at the district diabetes clinic was unreliable:

*“…The problem comes when you need medicines but you cannot find them… If you are in the village and run out… you cannot get them there....You must come here [town] to collect them….”* Female 07, 54 years

*“…For instance, last year diabetes medications disappeared for almost three weeks ….I could not find them anywhere in Ifakara ….and during that time I didn’t have a single medicine from the hospital…. That it is when you find us in these other places* (local healers/herbalists)*.”* Male 18, 56 years

In addition, participants expressed concerns about the structure and organization of diabetes services in the district. For example, participants reported that patients are advised to fast in preparation for fasting glucose measures, and to arrive at the clinic early for the tests. However, after reaching the facility, they often found that their consultation with the diabetes specialist was delayed as doctors made morning rounds in the wards before going to the diabetes clinic. Thus, participants reported having to wait six to eight hours for their consultation and prescription refill before eating. The inconvenience and discomfort associated with having to wait so long while hungry to access the services therefore appear to contribute to interruptions in medication use.

## Discussion

Our goal in this study was to explore lived experiences with diabetes medication use among adults living in rural areas. The participants were found to have positive perceptions of medications based on their efficacy, and to be aware that they need to take these medicines regularly. Thus, access to diabetes medications was shown to be the main barrier to use. The insufficient availability of diabetes medications [13,14] and their high costs [2,25] continue to make diabetes management in SSA, and in Tanzania in particular, [6,32,33], challenging. In the study area, diabetes medications often were not available, accessible, or affordable; especially for rural residents served by the primary health care facilities. There is a strong need to realign the health care services so that they cater to diabetes patients, as well as to those seeking episodic care.

The inclusion of some of the diabetes medicines in the national essential medicine list (NEMLIT) for Tanzania [34] is an indication that the government recognizes that these medicines are needed at primary-level facilities, and would be willing to improve the availability and the accessibility of diabetes medications. However, the inclusion of these medicines in the NEMLIT has so far not translated into improvements in drug supplies in health care facilities. This failure to ensure that facilities have adequate supplies of diabetes medicines forces many patients to access the medicines through the private sector, which imposes very heavy cost burdens on the patients and their families. To improve the availability and the accessibility of the diabetes medicines in the community, the government should take action to ensure the effective implementation of the NEMLIT.

Consistent adherence with the prescribed medication regimen is one of the critical factors in glycemic control [15,28] and the prevention of further clinical symptoms. In this study, participants reported that the high cost of clinical services and medications was the main reason why they sometimes failed to take their medications as prescribed. To delay having to refill their prescriptions, participants admitted they sometimes took a smaller dose than recommended or skipped a dose altogether. A similar pattern of failing to adhere to a medication regimen due to cost has also been observed among hypertension patients in other SSA countries [35,36]. The lack of adherence with medical recommendations may increase the risk of developing chronic complications [15]. Reductions of medication prices by pharmaceuticals could help to improve diabetes medication use and continuity. In addition, there is a need for ongoing health education campaigns which alert people with chronic, non-communicable conditions to the importance of taking their medications as recommended.

In line with observations made in other African settings [37,38], the study participants reported selling family assets to cover their medication costs. But in order to sell assets, a household has to have saleable property and access to potential buyers. The sale of assets to cover medical bills has been associated with the depletion of a household’s physical resources, and thus with an increased risk that the family will fall into poverty [24]. In turn, the consequences of poverty might even be greater for people with chronic conditions such as diabetes, who have additional expenses associated with long-term medication use. Ensuring that people have access to the health care services they need, and providing them with protection from financial risks, are steps toward ensuring universal health care coverage [39]. The current community health insurance schemes in Tanzania which aim to reduce direct out-of-pocket payments have so far mainly served people with episodic illnesses [40]. These schemes need to be strengthened to accommodate access to NCD health services, including access to medication. Other strategies based on social protection mechanisms—such as public subsidies for free care of the kind provided to patients with HIV/AIDS—could help to protect people with non-communicable diseases from financial risks, and encourage medication use and continuity of use.

Having social support from friends and family was shown to contribute to the resilience of the participants and to the likelihood that they were taking their medications as prescribed. These kinds of support with diabetes management appear to be associated with increased patient self-efficacy, medication regimen adherence, and better health outcomes [41,42]. It is therefore crucial to involve and educate household members in the management of diabetes care.

This study provides very important findings on experiences related to diabetes medication use and continuity of use from the perspectives of people living in rural areas where access to medications is limited. Recruiting study participants from the diabetes clinic allowed us to gain wider perspectives on experiences with continuous medication use from people in different rural areas. Although the study involved a particular group of people, it is likely that similar patterns of continuous medication use experiences exist in other rural parts of Tanzania and elsewhere in SSA, as inadequate availability of diabetes services and out-of-pocket payments are features of many SSA health systems [2,13,14]. The study findings can provide useful insights for developing strategies to improve and support access to diabetes care and treatments, to enhance self-management activities, and to encourage appropriate use and continuity of use of medications among patients.

## Conclusion

The study found that perceptions of diabetes medications were positive, but that the cost of the medications was a major barrier to their consistent use. Among the strategies the participants reported using to raise money to pay for diabetes medications were spending less money on family needs and selling household assets. A number of participants also reported sourcing their medicines from pharmacies to save on consultation and laboratory costs, asking family and friends for financial support, and borrowing cash. In addition, some participants admitted using less than the recommended dosage or skipping doses, and sharing medicines with other diabetes patients. However, these coping strategies may prove ineffective, and could compromise the health of the patient. More comprehensive strategies are therefore needed to encourage consistent medication use among people with chronic conditions. These strategies could include the reduction of prices by pharmaceuticals, the strengthening of community risk-pooling mechanisms (such as the introduction of a national or a social health insurance program, or of community health funds), and sustained health campaigns aimed at patients and the community.

## Endnote

^a^As all of the quotes have been translated from Swahili to English, they may not follow a strict grammatical route.

## Abbreviations

HIV/AIDS: Human immunodeficiency virus/acquired immune deficiency syndrome
IHI: Ifakara Health Institute
NCD: Non communicable disease
NIMR: National Institute for Medical Research
SSA: Sub Saharan Africa
